# Validation of “(fr)AGILE”: a quick tool to identify multidimensional frailty in the elderly

**DOI:** 10.1186/s12877-020-01788-1

**Published:** 2020-09-29

**Authors:** Ilaria Liguori, Gennaro Russo, Giulia Bulli, Francesco Curcio, Veronica Flocco, Gianlugi Galizia, David Della-Morte, Gaetano Gargiulo, Gianluca Testa, Francesco Cacciatore, Domenico Bonaduce, Pasquale Abete

**Affiliations:** 1 Department of Translational Medical Sciences, University of Naples “Federico II”, Via S. Pansini, 80131 Naples, Italy; 2 IRCCS Salvatore Maugeri Foundation, Scientific Institute of Veruno, Novara, Italy; 3 Department of Systems Medicine, University of Rome Tor Vergata, Rome, Italy; 4 San Raffaele Roma Open University, Rome, Italy; 5 Division of Internal Medicine, AOU San Giovanni di Dio e Ruggi di Aragona, Salerno, Italy; 6 Department of Medicine and Health Sciences, University of Molise, Campobasso, Italy

**Keywords:** Frailty, Multidimensional, Comprehensive geriatric assessment

## Abstract

**Background:**

Several tools have been proposed and validated to operationally define frailty. Recently, the Italian Frailty index (IFi), an Italian modified version of Frailty index, has been validated but its use in clinical practice is limited by long time of administration. Therefore, the aim of this study was to create and validate a quick version of the IFi (AGILE).

**Methods:**

Validation study was performed by administering IFi and AGILE, after a Comprehensive Geriatric Assessment (CGA) in 401 subjects aged 65 or over (77 ± 7 years). AGILE was a 10-items tool created starting from the more predictive items of the four domains of frailty investigated by IFi (mental, physical, socioeconomic and nutritional). AGILE scores were stratified in light, moderate and severe frailty. At 24 months of follow-up, death, disability (taking into account an increase in ADL lost ≥1 from the baseline) and hospitalization were considered. Area under curve (AUC) was evaluated for both IFi and AGILE.

**Results:**

Administration time was 9.5 ± 3.8 min for IFi administered after a CGA, and 2.4 ± 1.2 min for AGILE, regardless of CGA (*p* < 0.001). With increasing degree of frailty, prevalence of mortality increased progressively from 6.5 to 41.8% and from 9.0 to 33.3%, disability from 16.1 to 64.2% and from 22.1 to 59.8% and hospitalization from 17.2 to 58.7% and from 27.0 to 52.2% with AGILE and IFi, respectively (*p* = NS). Relative Risk for each unit of increase in AGILE was 56, 44 and 24% for mortality, disability and hospitalization, respectively and was lower for IFi (8, 7 and 4% for mortality, disability and hospitalization, respectively). The AUC was higher in AGILE vs. IFi for mortality (0.729 vs. 0.698), disability (0.715 vs. 0.682) and hospitalization (0.645 vs. 0.630).

**Conclusions:**

Our study shows that AGILE is a rapid and effective tool for screening multidimensional frailty, able to predict mortality, disability and hospitalization, especially useful in care settings that require reliable assessment instruments with short administration time.

## Background

Because of population aging, frailty has become a topic of growing interest in scientific and medical research [1–4].

Frailty does not yet have an internationally recognized standard definition, but it is considered a clinical condition characterized by an increased individual’s vulnerability in developing negative health-related events when exposed to potentially harmless stressors, due to age-related impairment in the four domains involved in individual’s health: physical, mental, socio-economic and nutritional [1].

Actually, around a quarter of people aged over 85 years are frail, thus they have an increased risk of developing adverse events including disability, hospitalization, institutionalization and death [2, 3]. In fact, the prevalence of frailty is dramatically increasing and questions about how it could be prevented and reliably detected are growing up [4].

The Comprehensive Geriatric Assessment (CGA) represents the reference methodological tool for frailty definition [5]. CGA is usually administered as part of clinical evaluation in the elderly to identify medical, functional and psychosocial needs through a holistic multidimensional assessment, highly predictive of adverse events also in different types of patients [6, 7]. However, despite the several clinical advantages of CGA, there may not be enough time and resources to perform it completely, especially in ambulatory settings in which most elderly outpatients are evaluated [8]. In addition, from a prognostic point of view, several specialties (i.e. surgery, oncology and cardiology) choose whether to perform several procedures according to patient’s prognosis, which can be established in elderly patients only estimating the degree of multidimensional frailty. Nevertheless, in these clinical settings, CGA cannot be used because of the lack of the time necessary to perform it [7, 9, 10].

Therefore, several tools have been proposed and validated to operationally define frailty and to predict its adverse outcomes, in terms of mortality, disability and hospitalization [11]. Among the most common frailty measurements, “Fried’s frailty phenotype” by Fried and colleagues and Frailty index (Fi) by Rockwood and Mitnitski are certainly the most widely used.

The “Fried’s frailty phenotype”, known as Cardiovascular Health Study (CHS) Index, investigates only the physical domain of frailty, which is defined by the presence of three or more of the following criteria: unintentional weight loss (≥4.5 Kg in the past year), self-reported exhaustion, weakness (grip strength), slow walking speed, and low physical activity [12].

The Fi is a cumulative estimate of potential health deficits which may occur with the aging process including symptoms, signs, diseases, disabilities or laboratory and/or instrumental abnormalities [13]. Fi is expressed as the ratio between the deficits found in a subject and the total number of deficits investigated. For these reasons, several studies have shown that Fi is more predictive of adverse clinical outcomes than other tools for both hospitalized and community dwelling older people [14, 15].

Thus, in our clinical practice, we have developed and recently validated an Italian modified version of Fi, the Italian Frailty index (IFi) [16]. However, despite its many advantages and reliability, IFi finds its greatest limitation in administration time. In fact, the time used for the administration of IFi alone is 40–50 min, compared to 9–15 min when it is derived from data already collected in a CGA [17].

However, Fi and IFi cannot be used in particular clinical setting because are both based on the results of a CGA, and therefore, require longer administration times [11].

Despite its clinical relief and the potential fields of application, an Italian version of a rapid tool for the identification of multidimensional frailty is not yet available. Therefore, the purpose of this study was to create and validate a rapid tool for evaluating multidimensional frailty.

## Methods

### Study population

Four hundred one subjects, aged ≥65 years, residents in Campania Region, non-disabled, clinically stable, Italian speaking, with life expectancy of at least 2 years were consecutively enrolled in the study from April 2014 to February 2017.

### CGA

The enrolled elderly subjects underwent a CGA that investigated the following aspects: cognitive function with the Mini Mental State Examination (MMSE); mood symptomatology with the Geriatric Depression Scale (GDS); comorbidity severity with the Cumulative Illness Rating Scale (CIRS); total number of drugs taken; disability with Basic and Instrumental Activities of Daily Living (BADL and IADL); nutritional grade with the Mini Nutritional Assessment (MNA); gait, balance and risk of falls with the Tinetti Scale and the Short Physical Performance Battery (SPPB); physical activity with the Physical Activity Scale for the Elderly (PASE) and the degree of social support with the Social Support Score (SSS) [18]. In addition, all the enrolled subjects were evaluated for orthostatic hypotension [19].

### IFi

As previously mentioned, the IFi is an Italian modified version of the Fi recently validated on a cohort of 1077 non-disabled outpatients aged ≥65 years (81.3 ± 6.5 years) (Abete2017). Similar to Fi, IFi includes 40 items, corresponding to as many functional deficits. However, to better define the socio-economic and nutritional frailty, the IFi differs from the original Fi in the item #24 (“Feeling lonely”) and in the item #39 (“Time taken to walk four meters with habitual step”), which have been substituted by SSS score (> 13 = 1; 6–13 = 0.5 and 1–5 = 0) and at the MNA (< 17 = 1; 17–23 = 0.5 and 24 = 0), respectively. The IFi was expressed as the amount of the scores obtained to each of the 40 items examined by the tool. Then, the results obtained by administering IFi were divided into tertiles: light frailty (0.0–16.0), moderate frailty (16.1–27) and severe frailty (27.0–40.0).

### Agile

AGILE is a frailty tool coming from the growing need to quickly identify frail elderly, but also to investigate “multidimensional” frailty. Thus, AGILE was built by selecting among the 40 items of IFi the 10 ones most predictive of mortality, in order to homogeneously represent the four domains of “multidimensional” frailty: physical, mental, nutritional and socio-economic (Additional file 1 - items in bold).

Thus, the items were selected as following (Table 1):

- Physical frailty: (1) “Feel everything is an effort” (#21 of IFi), (2) “Help up/down stairs” (#8 of IFi), (3) “Grip strength” (#38 of IFi), calculated by the mean of three measurements made using a Jamar® hydraulic dynamometer;
- Mental frailty: deficits in two MMSE items (#34 of the IFi): (4) temporal orientation (date/month/year) and (5) delayed recall of the three words “bread-house-cat” (referred to the patient at the beginning of the questionnaire); (6) Feel depressed (#22 of IFi);
- Nutritional frailty: (7) “Weight loss over 4.5 Kg in the last year” (#15 of IFi); (8) “Help in eating” (#5 of IFi).
- Socio-economic frailty: using two items of the SSS (#24 of the IFi): (9) “Financial help from family members” and (10) “Physical help from family members”.

**Table 1:** The 10 items of AGILE tool with relative scoring system divided by domain of frailty (physical, mental, nutritional and socio-economic)

n.	Item	Score	Frailty domain
**1**	Feel everything is an effort	yes = 1; no = 0	**Physical**
**2**	Help up/down stairs	yes = 1; no = 0	
**3**	Grip strength^(1)^	yes = 1; no = 0	
**4**	Temporal orientation deficit^(2)^	yes = 1; no = 0	**Mental**
**5**	Delayed recall deficit^(3)^	yes = 1; no = 0	
**6**	Feel depressed	yes = 1; no = 0	
**7**	Weight loss over 4.5 kg in the last year	yes = 1; no = 0	**Nutritional**
**8**	Help in eating	yes = 1; no = 0	
**9**	Financial help from family members	yes = 0; no = 1	**Socio-economic**
**10**	Physical help from family members	yes = 0; no = 1	

^(1)^≤30 Kg in men, ≤20 Kg in women at hand-held dynamometer

^(2)^The subject does not refer the exact date (day/month/year)

^(3)^The words “bread-house-cat” are referred to the subject at the beginning of the questionnaire and then asked to the subject at this time of the questionnaire

**Fig. 1:**
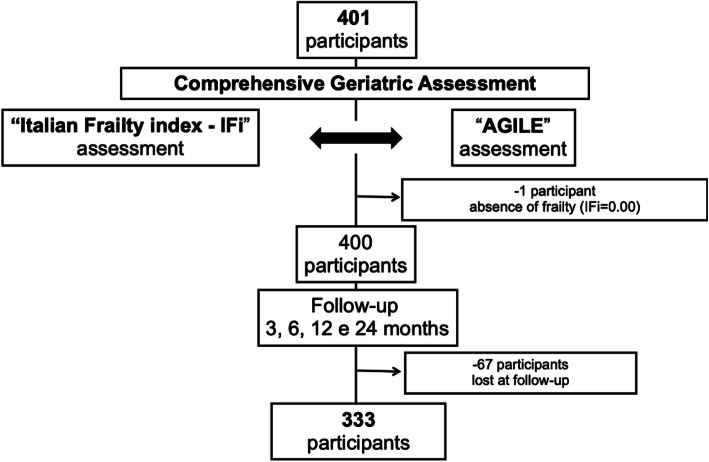
Study protocol flow chart

All the binary variables have been coded using “0” to indicate the absence and “1” the presence of a deficit, except for the two items selected from the SSS where in the original scoring system the absence of help corresponds to “1” and the presence of help to “0”. The AGILE scores were then divided into tertiles: light (0–3), moderate (4–7) and severe frailty (8–10). Among the 401 elderly subjects who received IFi, only 1 obtained a score of 0.00, and for this reason it was excluded from the study. Then, 400 subjects were included in the follow-up at 24 months and 333 of them (83.2%) completed the study (Fig. 1).

### Outcomes

At 24 months of follow-up, death, disability (taking into account an increase in ADL lost ≥1 from the baseline) and hospitalization. Follow-up was performed also at 3, 6, 12 and 18 months but these data are not reported in this paper because they were incomplete.

### Statistical analysis

Continuous variables are shown as mean ± standard deviation (SD) while categorical variables as percentages, respectively. Continuous variables across groups was analyzed by ANOVA test and corrected by Bonferroni’s post hoc test while and Chi-square test was used to evaluate categorical variables. AGILE’s inter-rater reliability was evaluated we calculated in 25 subjects by using a kappa coefficient assuming that excellent agreement was indicated by a value of ≥0.80 while Crohnbach’s value was performed to compare internal reliability. Associations between frailty, defined according both to AGILE and IFi, and subsequent outcomes, including mortality, disability and hospitalization was evaluated by logistic multivariate analysis adjusted for age and sex; Relative Risk (RR) of each outcome, with 95% confidence intervals, was calculated per unit of increase of both AGILE and IFi. In order to compare the sensitivity and specificity of AGILE vs. IFi in predicting the outcomes, Receiver Operating Characteristics (ROC) curves were analyzed by logistic regression analysis; Area Under Curve (AUC) between 0.7 and 0.9 was considered significant. Finally, in this study we use Lin’s Concordance Correlation Coefficient, a method currently used to compare a new measurement method (AGILE) with the standard method (IFi) [20]. The sample size was 350 subjects with a study power of 0.90 (one-sided z test with α = 0.05) with a CCC value between 0.80–0.75.

## Results

**Fig. 2:**
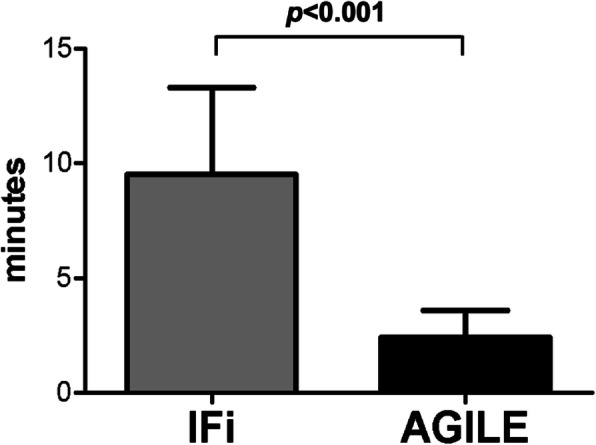
Time of administration of the Italian Frailty index (IFi) and AGILE. It should be noted that IFi was administered after the Comprehensive Geriatric Assessment while AGILE without the Comprehensive Geriatric Assessment

The flow chart of the study with the final number of participants is shown in Fig. 1. The IFi and AGILE showed a good inter-rater variability (k = 0.84, *p* < 0.001; *n* = 25 for IFi; k = 0.86, *p* < 0.001; *n* = 25 for AGILE) and good internal consistency (Crohnbach’s value for IFi = 0.83 and for AGILE = 0.87). Administration time for IFi alone was 40.6 ± 9.8 min compared to 9.5 ± 3.8 min when it is derived from data already collected in a CGA. Administration time for AGILE was 2.4 ± 1.2 min regardless of the CGA (*p* < 0.001 vs. IFi) (Fig. 2).

Demographic characteristics and CGA scores of the cohort are described in Table 2. Mean age was 77 ± 7 years. Female subjects were 55.5% of the sample (*n* = 222). In the study cohort, IFi reached a score of 30.4 ± 2.1, while AGILE 8.3 ± 0.5 in the highest frailty degree. Regarding the variables examined in the CGA, the increase in frailty degree, from light to severe, according to AGILE, corresponded to an increase in comorbidity (from 1.6 ± 0.4 to 2.0 ± 0.6; *p* < 0.001), number of drugs taken (from 4.7 ± 3.3 to 7.4 ± 2.9; *p* < 0.001), in BADL (from 0.5 ± 0.9 to 3.8 ± 1.3; *p* < 0.001) and IADL lost (from 1.4 ± 2.0 to 6.5 ± 1.9; *p* < 0.001). At the same time, the increase in frailty degree, according to AGILE, corresponded to a reduction in MMSE score, reaching a value of 15.0 ± 6.2 (*p* < 0.001), and worse nutritional indices, such as BMI and MNA score, as well as in the Tinetti, SPPB and PASE scores. Moreover, the increase in the frailty degree according to AGILE is also associated to a progressive worsening in social support, corresponding to increasing SSS scores (from 6.0 ± 3.3 to 9.7 ± 2.2 *p* < 0.001).

**Table 2:** Demographic characteristics and Comprehensive Geriatric Assessment of the study sample stratified for frailty degree

Variables	All (n. 400)	Light (n.72)	Moderate (n.260)	Severe (n.68)	***p*** per trend
Age (years)	77 ± 7	74 ± 6	77 ± 7	80 ± 6	0.001
Sex (% female)	55.5 (222)	34.7 (25)	56.5 (147)	73.5 (50)	0.001
BMI	27 ± 5	27 ± 5	28 ± 6	26 ± 5	0.183
Systolic BP, (mmHg)	141 ± 23	134 ± 20	142 ± 23	147 ± 26	0.019
Diastolic BP, (mmHg)	80 ± 13	78 ± 10	80 ± 11	83 ± 13	0.163
Orthostatic hypotension (n,%)	14.2 (34.2)	15.1 (8)	14.7 (23)	9.7 (3)	0.743
CIRS	1.8 ± 0.5	1.6 ± 0.4	1.8 ± 0.5	2.0 ± 0.6	0.001
Drugs number	6.1 ± 3.2	4.7 ± 3.3	6.1 ± 3.1	7.4 ± 2.9	0.001
BADL lost	2.2 ± 1.8	0.5 ± 0.9	2.2 ± 1.7	3.8 ± 1.3	0.001
IADL lost	4.3 ± 2.9	1.4 ± 2.0	4.6 ± 2.7	6.5 ± 1.9	0.001
MMSE, score	20.8 ± 6.6	26.3 ± 3.1	20.8 ± 6.1	15.0 ± 6.2	0.001
GDS, score	8.0 ± 4.1	3.7 ± 3.1	8.4 ± 3.7	10.6 ± 3.4	0.001
MNA, score	21.8 ± 11.1	26.0 ± 2.3	21.9 ± 13.3	17.3 ± 2.8	0.001
Tinetti, score	17.3 ± 8.1	25.3 ± 3.5	19.9 ± 7.6	10.4 ± 5.9	0.001
PASE, score	32.6 ± 52.7	77.7 ± 67.0	25.1 ± 39.8	15.1 ± 56.2	0.001
SPPB, score	5.6 ± 3.9	8.7 ± 3.1	5.3 ± 3.6	2.3 ± 2.8	0.001
Social Support Score	8.5 ± 2.9	6.0 ± 3.3	8.8 ± 2.6	9.7 ± 2.2	0.001
**IFi**	20.0 ± 9.0	9.4 ± 3.9	22.4 ± 3.0	30.4 ± 2.1	0.001
**AGILE**	5.5 ± 2.0	2.4 ± 0.6	5.6 ± 1.0	8.3 ± 0.5	0.001

*BMI* Body Mass Index, *BP* blood pressure, *CIRS* Cumulative Illness Rating Scale, *BADL* Basic Activity of Daily Living, *IADL* Instrumental Activity Daily Living, *MMSE* Mini Mental State Examination, *GDS* Geriatric Depression Scale, *MNA* Mini Nutritional Assessment, *SPPB* Short Physical Performance Battery, *PASE* Physical Activity Scale for the Elderly, *IFi* Italian Frailty index

The prevalence of frailty of the study sample for AGILE items stratified for frailty degree and domain of multidimensional frailty are listed in Table 3. As expected, more severe frailty degrees corresponded to higher percentage of positivity for each of the 10 items. In particular, a percentage of 100% in the highest percentages was found in the items exploring the physical domain of frailty: “Feel everything is an effort” and “Grip strength”.

**Table 3:** Prevalence of frailty of the study sample for AGILE items stratified for frailty degree and domain of multidimensional frailty

Item	All (n.400)	Light (n.72)	Moderate (n.260)	Severe (n.68)	***p*** per trend
	(%, n.)	(%, n.)	(%, n.)	(%, n.)	
**Physical**					
Help up/down stairs	51.8 (207)	1.4 (1)	53.5 (139)	98.5 (67)	0.001
Feel everything is an effort	83.8 (335)	43.1 (31)	90.8 (236)	100.0 (68)	0.001
Grip Strength	80.8 (323)	33.3 (24)	88.8 (231)	100.0 (68)	0.001
**Mental**					
Temporal orientation deficit at MMSE	55.0 (220)	12.5 (9)	56.5 (147)	94.1 (64)	0.001
Delayed recall deficit at MMSE	80.8 (323)	51.4 (37)	84.2 (219)	98.5 (67)	0.001
Feel depressed	75.8 (303)	26.4 (19)	83.8 (218)	97.1 (66)	0.001
**Nutritional**					
Lost more than 4.5 Kg in last year	29.3 (117)	12.5 (9)	24.2 (63)	66.2 (45)	0.001
Help eating	23.5 (94)	0.0 (0)	16.9 (44)	73.5 (50)	0.001
**Socio-economic**					
Financial help from family members	52.8 (211)	44.4 (32)	42.2 (76)	69.6 (103)	0.001
Physical help from family members	22.0 (88)	18.1 (13)	19.4 (35)	25.0 (37)	0.181

*MMSE* Mini Mental State Examination

Multivariate analysis, performed for each of the 10 items of the AGILE, showed that the most predictive items for the three study outcomes were: “Help eating” (3.48, CI 95% 1.01–6.26; *p* < 0.001) for mortality, “Delayed recall deficit at MMSE” (RR 3.40, CI 95% 1.75–6.61; *p* < 0.001) for disability and “Feel everything is an effort” (RR 2.38; CI 95% 1.16–4.85; *p* = 0.017) for hospitalization (Table 4).

**Table 4:** Multivariate analysis, adjusted for age and sex, on mortality, disability and hospitalization of AGILE items for each domain of multidimensional frailty

Events	Mortality			Disability			Hospitalization		
	RR	95% CI	***p***	RR	95% CI	***p***	RR	95% CI	***p***
**Physical**									
Help up/down stairs	3.08	1.62–5.87	0.001	3.36	2.08–5.42	0.001	1.81	1.12–2.94	0.015
Feel everything is an effort	3.31	1.16–9.64	0.028	1.84	0.97–3.49	0.061	2.38	1.16–4.85	0.017
Grip Strength	1.46	0.67–3.18	0.332	2.09	1.14–3.81	0.016	0.78	0.44–1.39	0.413
**Mental**									
Temporal orientation deficit at MMSE	2.31	1.23–4.35	0.009	2.97	1.84–4.78	0.001	1.58	0.97–2.55	0.061
Delayed recall deficit at MMSE	2.90	1.11–7.61	0.029	3.40	1.75–6.61	0.001	2.07	1.08–3.95	0.027
Feel depressed	1.92	0.91–4.05	0.086	1.88	1.09–3.25	0.023	1.19	0.69–2.07	0.516
**Nutritional**									
Lost more than 4.5 Kg in last year	2.21	1.26–3.87	0.005	1.30	0.81–2.09	0.266	1.42	0.88–2.29	0.148
Help eating	3.48	1.01–6.26	0.001	1.49	0.89–2.50	0.125	1.76	1.05–2.95	0.031
**Socio-economic**									
Financial help from family members	1.86	1.06–3.26	0.029	2.20	1.41–3.43	0.001	1.76	1.12–2.76	0.014
Physical help from family members	0.84	0.43–1.65	0.847	0.64	0.38–1.10	1.108	0.96	0.56–1.64	0.895

RR Relative Risk, *CI* Confidence Interval, *MMSE* Mini Mental State Examination

**Fig. 3:**
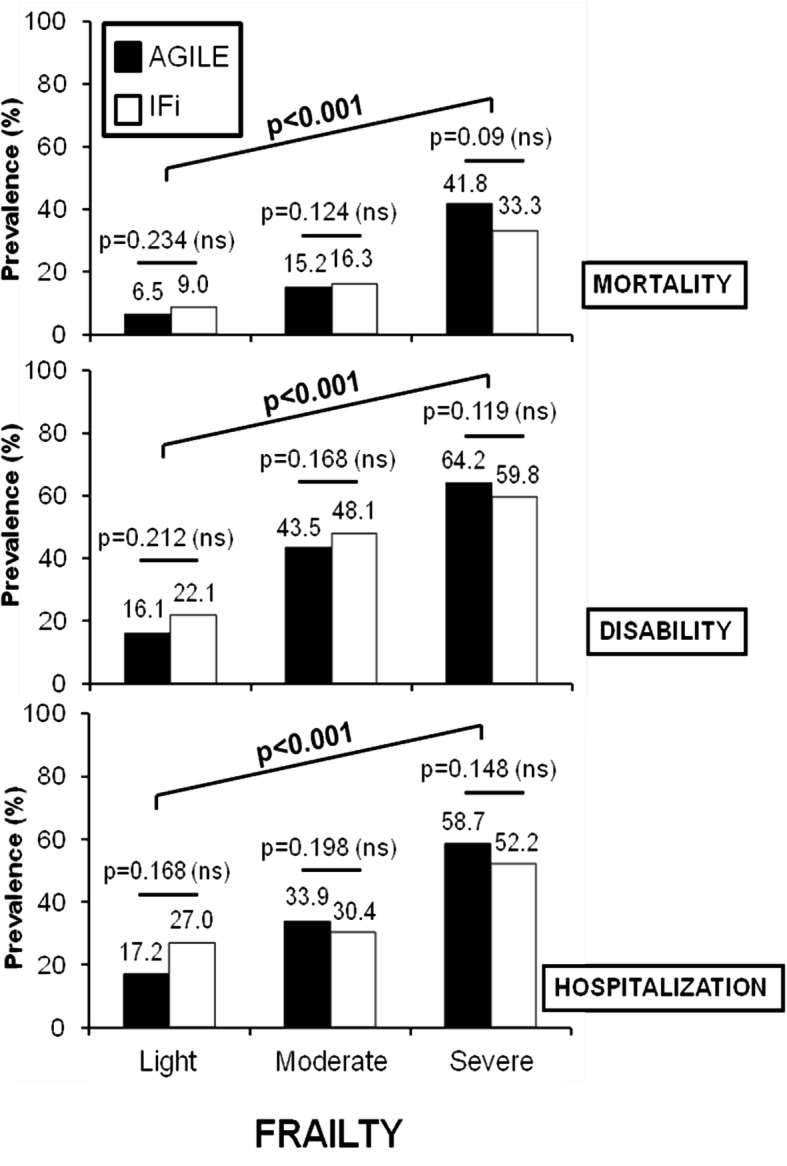
Prevalence of mortality, disability (considering an increase in ADL lost ≥1 from the baseline) and hospitalization at 24 months stratified by light, moderate and severe frailty according to AGILE and IFi (Italian Frailty index)

In Fig. 3 are reported the prevalence of mortality, disability (considering an increase in ADL lost ≥1 from the baseline) and hospitalization. At the end of the follow-up (24 months), mortality increased progressively from 6.5 to 41.8% with AGILE and from 9.0 to 33.3% with IFi (*p* = NS), the disability showed a progressive increase from 16.1 to 64.2% with AGILE and from 22.1 to 59.8% with IFi (*p* = NS) and hospitalization gradually increased from 17.2 to 58.7% with AGILE and from 27.0 to 52.2% with IFi (*p* = NS).

Multivariate logistic regression analysis, adjusted for age and sex, showed that the RR for each unit of increase in AGILE was 56, 44 and 24% for mortality, disability and hospitalization, respectively. As expected, RR increase for each unit of IFi was lowers (8, 7 and 4% for mortality, disability and hospitalization, respectively) because IFi score may reach a maximum value of 40 while AGILE only of 10 (Table 5).

**Table 5:** Multivariate analysis, adjusted for age and sex, on mortality, disability and hospitalization of AGILE and IFi for each unit of increase

Events	RR	95% CI	***p***
**Mortality**			
AGILE	1.56	1.31–1.86	< 0.001
IFi	1.08	1.04–1.12	< 0.001
**Disability**			
AGILE	1.44	1.26–1.64	< 0.001
IFi	1.07	1.04–1.10	< 0.001
**Hospitalization**			
AGILE	1.24	1.09–1.40	< 0.001
IFi	1.04	1.01–1.07	0.004

*IFi* Italian Frailty index), *RR* Relative Risk for each unit of increase of AGILE or IFi score, *CI* Confidence Interval

**Fig. 4:**
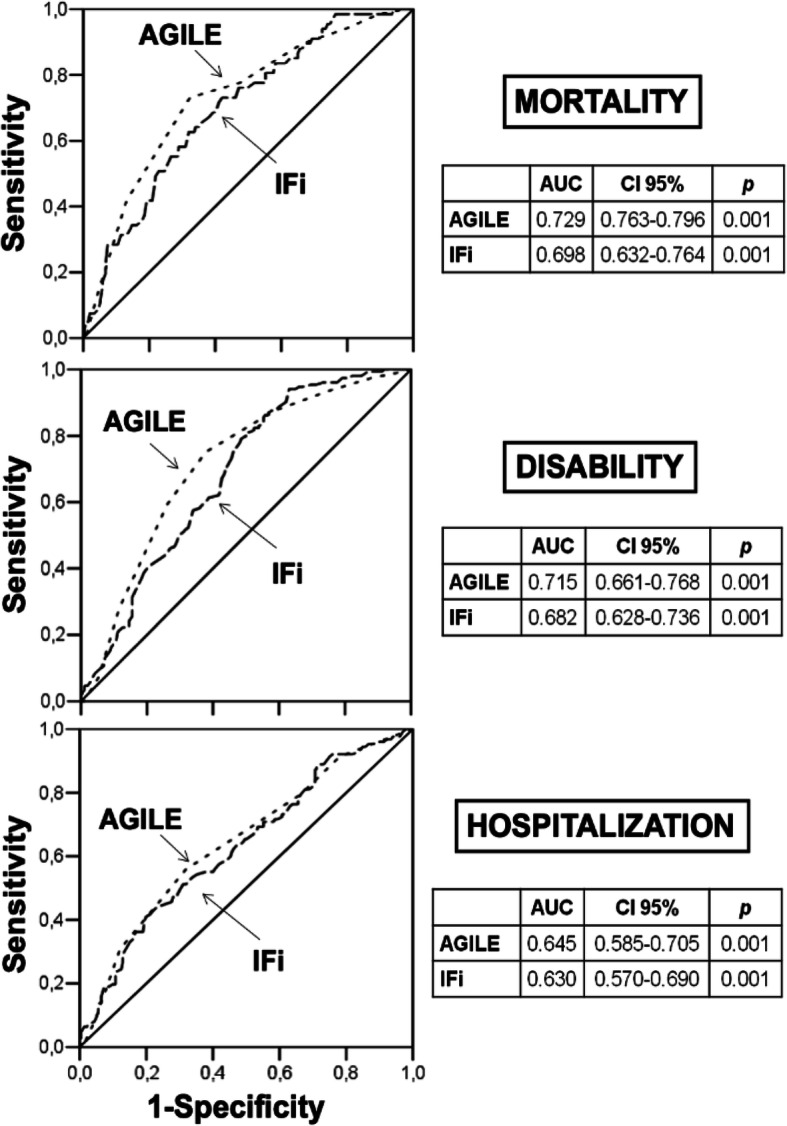
ROC (Receiver Operating Characteristics) curves of AGILE and IFi (Italian Frailty index) on mortality, disability and hospitalization

In Fig. 4 are represented the ROC curves of AGILE vs. IFi for mortality, disability and hospitalization. AGILE and IFi show a similar predictive value for each adverse clinical event and the AUC is unexpectedly higher in AGILE than IFi for all the study outcomes.

## Discussion

Our results suggest that AGILE is a quick and reliable tool for multidimensional frailty evaluation. AGILE tool allows, in a very short time (≈2.5 min), to stratify subjects into different frailty degrees: not-frail (score 0), light (score 1–3), moderate (score 4–7) or severe (score 8–10) and to investigate the four domains of multidimensional frailty: physical, mental, socioeconomic and nutritional.

Because of these characteristics, AGILE differs from many of the frailty measurement tools available in literature:

- CHS Index despite its widespread use investigates only the physical domain of frailty [12], while AGILE investigates also the mental, socio-economic and nutritional ones.
- Fi although thoroughly investigates all frailty domains, can be time consuming to perform and calculate and becomes time-efficient only when derived from data already collected in a CGA [13]; AGILE does not have this limitation and does not need to be administered after a CGA.
- The Study of Osteoporotic Fractures (SOF) index investigates physical frailty above all and despite its reliability [21] does not investigate multidimensional frailty and tend to over-screen frailty in the hospital setting because patients often cannot perform a five-times-chair-rise. AGILE not only investigates frailty from a multidimensional point of view but lacks of this latter limitation because the items chosen to investigate physical frailty (“Feel everything is an effort”, “Help up/down stairs” and “Grip strength” measured by dynamometer) can be without difficulty administered also in hospital setting.
- The FRAIL (Fatigue, Resistance, Ambulation, Illness, Loss of Weight) Index, recently proposed by the International Association of Nutrition and Ageing [22], is judged to be clinically advantageous due to its simple nature but, compared to AGILE, does not investigate socioeconomic and mental domains of frailty.
- The Tilburg Frailty Indicator (TFI) encompasses physical components (health, weight loss, difficulty in walking, balance, hearing, vision, gripping and tiredness), psychological factors (memory, feeling down, anxiety and coping) and social elements (living alone, social isolation, social support), but only its physical components shows a good predictive value unlike its social one [11, 23]. Conversely, in AGILE only one of the two items investigating socioeconomic domain (“Physical help from family members”) does not show predictive value (Table 4), probably due the fact that enrolled subjects were community dwelling and were often accompanied by a caregiver, showing higher degree of social support.
- PRISMA-7 contains seven simple components to identify frailty: age ≥ 85 years; male sex; physical problems which reduce daily activities; another person help required; wellbeing problems obliging to reside at home; social support; and use of a cane/walker/wheelchair [24]. This tool, compared to AGILE, has a tendency to over-screen for frailty and does not investigate nutritional domain of frailty.

In fact, interestingly, in our study AUC is higher in AGILE than IFi. In validation study, AUC of IFi were 0.809 for mortality, 0.800 for disability and 0.707 for hospitalization, and these values are higher to those observed in the present study. One explanation should be the different sample size of IFi original validation study which included 907 participants and the different clinical characteristics between the two validation studies [16]. Anyway, AGILE leads to better results probably because it is an easier and faster tool to administer than IFi and these characteristics probably reduce the risk of administration and/or compilation mistakes.

Because of the rapid increase in frailty prevalence, early recognition of this clinical condition in the elderly has become mandatory in order to avoid the risk to treat patients only on the basis of their medical conditions without taking into account their overall frailty status [11]. Including an instrument like AGILE into clinical practice may provide a tool for clinicians to early identify and manage this condition in order to prevent adverse clinical outcomes. Frailty is an emerging global health burden and several studies have shown how increased health-care costs and use are associated with prevalent frailty or a higher degree of frailty [25]. The idea to organize the 10 items of AGILE according to frailty domains derives from the need to briefly identify the more compromised domain (“risk domain”) to develop personalized intervention and prevention strategies. In fact, several interventions have been proposed to prevent, delay or reverse frailty in older people, such as physical exercise, nutritional supplementation, cognitive training and combined treatment [26]. Furthermore, several studies have also established the effectiveness of tailored management programs to prevent frailty progression [27, 28].

### Study limitations

Our study is not a multicenter study and therefore, not including participants from different geographical areas, the results obtained could only be partially representative and valid for the entire Italian population. Therefore, further studies will be needed to evaluate the reliability and reproducibility of this tool in other settings and populations. The enrolled subjects are community dwelling, probably leading to a selection *bias* related to the severity comorbidities and degree of social support. In fact, subjects with severe degrees of disability are often institutionalized and the elderly subjects enrolled in our ambulatory services were often accompanied by a caregiver, showing higher degree of social support.

## Conclusions

Our findings suggest that AGILE is a quick and reliable tool for multidimensional frailty with a high predictive value of adverse clinical outcomes, such as mortality, disability and hospitalization. Due to the short administration time and the ability to identify the most compromised domains of multidimensional frailty, AGILE represents a suitable tool to target preventive therapeutic strategies, especially in the care settings where administration time is a limiting factor.

## Supplementary information

**Additional file 1.** Relative risk and ranking weight of the 40 items of frailty index on mortality, disability (≥ 1 ADL lost) and hospitalization

## Data Availability

The data supporting this article can be made available from the corresponding author on reasonable request.

## Abbreviations

AUC: Area Under Curve
BADL: Basic Activities of Daily Living
CGA: Comprehensive Geriatric Assessment
CHS: Cardiovascular Health Study
CIRS: Cumulative Illness Rating Scale
FRAIL: Fatigue, Resistance, Ambulation, Illness, Loss of Weight
GDS: Geriatric Depression Scale
IADL: Instrumental Activities of Daily Living
IFi: Italian Frailty index
MMSE: Mini Mental State Examination
MNA: Mini Nutritional Assessment
PASE: Physical Activity Scale for the Elderly
ROC: Receiver Operating Characteristics
RR: Relative Risk
SD: Standard Deviation standard deviation
SOF: Study of Osteoporotic Fractures
SPPB: Short Physical Performance Battery
SSS: Social Support Score
TFI: Tilburg Frailty Indicator
