# Supplementary material for: Validation of “(fr)AGILE”: a quick tool to identify multidimensional frailty in the elderly
Source: BMC Geriatr. 2020 Sep 29;20:375. doi: 10.1186/s12877-020-01788-1 (PMC7526099; doi:10.1186/s12877-020-01788-1)
Supplement: Supplementary file 1 — Additional file 1. Relative risk and ranking weight of the 40 items of frailty index on mortality, disability (≥ 1 ADL lost) and hospitalization [file 12877_2020_1788_MOESM1_ESM.doc]

**Appendix 1**: Relative risk and ranking weight of the 40 items of frailty index on mortality, disability (≥ 1 ADL lost) and hospitalization.

| ITEMS | Mortality | | |
| --- | --- | --- | --- |
|  | **RR** | **CI 95%** | **p** |
| 1. Help bathing | 0.98 | 0.32-1.14 | 0.08 |
| 1. Help dressing | 2.69 | 0.73-9.88 | 0.14 |
| 1. Help getting in/out of chair | 2.42 | 1.20-6.62 | 0.02 |
| 1. Help walking around house | 2.07 | 0.95-4.49 | 0.07 |
| 1. Help eating | **2.02** | **1.00-4.08** | **0.05** |
| 1. Help grooming | 0.97 | 0.31-2.97 | 0.95 |
| 1. Help using toilet | 1.78 | 0.81-3.93 | 0.15 |
| 1. Help up/down stairs | **4.12** | **1.21-14.0** | **0.02** |
| 1. Help lifting 10 lbs | 1.83 | 0.85-3.94 | 0.12 |
| 1. Help shopping | 0.71 | 0.23-2.21 | 0.55 |
| 1. Help with housework | 0.66 | 0.29-1.50 | 0.33 |
| 1. Help with meal preparations | 0.96 | 0.38-2.47 | 0.94 |
| 1. Help taking medication | 0.69 | 0.32-1.52 | 0.36 |
| 1. Help with finances | 0.89 | 0.41-1.93 | 0.76 |
| 1. Lost more than 10 lbs in last year | **2.28** | **1.31-3.94** | **0.00** |
| 1. Self rating of health | 0.12 | 0.03-0.56 | 0.01 |
| 1. Health has changed in last year | 1.39 | 0.60-3.21 | 0.44 |
| 1. Stayed in bed at least half the day due to health | 2.38 | 1.29-4.41 | 0.01 |
| 1. Cut down on usual activity | 1.85 | 0.89-3.85 | 0.10 |
| 1. Walk outside | 0.92 | 0.42-2.01 | 0.84 |
| 1. Feel everything is an effort | **4.83** | **1.33-17.5** | **0.02** |
| 1. Feel depressed | **1.17** | **1.05-2.24** | **0.04** |
| 1. Feel unhappy | 0.56 | 0.20-1.60 | 0.28 |
| 1. Social support | **2.30** | **1.00-5.31** | **0.05** |
| 1. Have Trouble getting going | 0.39 | 0.15-1.02 | 0.05 |
| 1. High blood pressure | 0.88 | 0.44-1.74 | 0.71 |
| 1. Heart attack | 1.19 | 0.61-2.32 | 0.61 |
| 1. CHF | 2.25 | 1.17-4.33 | 0.02 |
| 1. Stroke | 0.97 | 0.45-2.13 | 0.95 |
| 1. Cancer | 2.93 | 1.61-5.35 | 0.00 |
| 1. Diabetes | 0.93 | 0.53-1.65 | 0.80 |
| 1. Arthritis | 0.63 | 0.32-1.22 | 0.17 |
| 1. Chronic Lung Disease | 1.18 | 0.65-2.13 | 0.58 |
| 1. MMSE | **1.02** | **1.01-1.99** | **0.02** |
| 1. BMI | 0.85 | 0.44-1.64 | 0.63 |
| 1. Peak Expiratory Flow | 2.42 | 1.10-5.21 | 0.03 |
| 1. Shoulder Strength | 0.54 | 0.27-1.08 | 0.08 |
| 1. Grip Strength | **2.56** | **1.13-5.82** | **0.02** |
| 1. Mini Nutritional Assessment | 0.72 | 0.33-1.57 | 0.41 |
| 1. Rapid Pace | 2.00 | 1.06-3.80 | 0.03 |

Legend: RR=relative risk; CI=Confidence interval
